# Acute rhabdomyolysis in hepatitis-associated aplastic anemia patient undergoing allogeneic hematopoietic stem-cell transplantation: case report and literature review

**DOI:** 10.1186/s40001-022-00675-2

**Published:** 2022-03-21

**Authors:** Yuzhu Li, Yilei Hong, Yingying Shen, Qi Liu, Ying Chen, Keding Shao, Yiping Shen, Baodong Ye, Dijiong Wu

**Affiliations:** 1 The First School of Clinical Medicine, Zhejiang Chinese Medical University, Hangzhou, Zhejiang China; 2 Department of Hematology, The First Affiliated Hospital of Zhejiang Chinese Medical University, Hangzhou, 310006 Zhejiang People’s Republic of China; 3 Office of Academic Research, Zhejiang Chinese Medical University, Hangzhou, Zhejiang China

**Keywords:** Anemia, aplastic, Hepatitis, Rhabdomyolysis, Hematopoietic stem cell transplantation

## Abstract

**Background:**

Hepatitis-associated aplastic anemia (HAAA) is a specific type of aplastic anemia, and hematopoietic stem-cell transplantation (HSCT) is recommended as the first-line. Acute rhabdomyolysis (AR) during hematopoietic stem-cell transplantation (HSCT) is a rare, serious complication, with only 10 cases reported in the world so far.

**Case presentation:**

Herein, we present a case of AR developing during HLA-haploidentical HSCT in a 55-year-old man who suffered from HAAA. On day 7 after stem cell transfusion, the patient reported a muscle pull in thigh and complained of muscle swelling, pain and change in urine color. Despite the timely diagnosis (based on the levels of myoglobin and creatine kinase, and muscle MRI findings, etc.) and rapid hydration and alkalization, the situation progressed dramatically, and the patient died of multi-organ failure during the preparation for continuous renal replacement therapy (CRRT). Five days after his death, the whole-exome sequencing result confirmed that the patient had a germline missense mutation in SCN4A I 1545 V and ACTN3 R577X.

**Conclusion:**

AR is a rare but threatening complication during HSCT, especially in cases with kidney dysfunction. The creatine kinase level may not truly and completely reflect the severity and prognosis for cases with localized lesion. We suggest that genetic analysis should be performed for better understanding the pathological changes of AR during HSCT, especially for patients with bone marrow failure.

## Background

Aplastic anemia (AA) is a disorder characterized by bone marrow hematopoietic failure and pancytopenia [1]. Hepatitis-associated aplastic anemia (HAAA) is a specific type of AA, manifested as pancytopenia within 6 months after the onset of acute hepatitis [2]. Compared to acquired idiopathic AA, the degree of bone marrow failure in HAAA is often more obvious, manifested mainly as severe aplastic anemia (SAA), very severe aplastic anemia (VSAA), or even fulminant aplastic anemia (FAA). Hematopoietic stem-cell transplantation (HSCT) is a crucial treatment for AA, especially for SAA, recommended as the first-line treatment for patients with HAAA [1, 2]. In spite of its high cure rate, HSCT has its limitations and complications, including infections, sinus occlusion syndrome (SOS), graft-versus-host disease (GVHD), and engraftment failure, which may lead to transplant failure and even death [3]. With the development of transplantation technology, the prognosis of AA undergoing HSCT has considerably improved. However, some rare but severe complications during HSCT may still threaten the life of the patient.

Rhabdomyolysis is destruction of muscle cells caused by a variety of factors, resulting in the release of intracellular substances into the extracellular fluid. This condition is typically characterized by myalgia, muscle weakness, and dark urine, and has tripled mortality rates when combined with acute renal injury [4]. Herein, we report a case of failed treatment of acute rhabdomyolysis (AR) during HSCT further confirmed to have germline SCN4A I1545V and ACTN3 R577X missense mutations. To better understand this rare complication, we further reviewed the available evidence of reported cases, analyzed the clinical features, probable risk factors, and prognosis, as well as the genetic susceptibility to this disorder of patients undergoing HSCT, which may facilitate earlier diagnoses and timely rescue from this life-threatening disorder.

## Case report

A 55-year-old Asian male was admitted on September 2020 to our clinic with a complaint of fatigue and ecchymosis for 10 days. On admission, his routine blood test results were as follows: white blood cell (WBC) 1.0 × 10^9^/L, absolute neutrophil count (ANC) 0.4 × 10^9^/L, hemoglobin (HB) 80 g/L, platelet (PLT) count 11 × 10^9^/L, reticulocyte (Ret) 13.64 × 10^9^/L. Ilium and sternum bone marrow aspiration showed bone marrow hematopoietic failure, and the proportion of lymphocytes accounted for 71.5%. There was no evidence of myelofibrosis or dysmorphic hematopoiesis, and cytogenetic examination revealed a normal male karyotype. The liver biochemical parameters were normal, and the serological indicators suggested negative results for hepatitis viruses A, B, C, and E, as well as for Epstein–Barr virus and cytomegalovirus. The antibody test for autoimmune hepatitis was weakly positive for anti-smooth muscle antibodies. Two months ago, he had been admitted to another hospital because of calf edema. At that time, aspartate aminotransferase (AST, 143 U/L) and alanine aminotransferase (ALT, 177 U/L) were abnormally high, whereas the routine blood test results were normal. The diagnosis of HAAA was confirmed considering the history of hepatitis, along with the differential diagnosis from other diseases manifested with pancytopenia (such as acute arrest of hemopoiesis, myelodysplastic syndrome, paroxysmal nocturnal hemoglobinuria, and leukemia). The patient had no improvement after intravenous immunoglobulin (IVIg, 20 g per day for 5 days), and considering no available sibling-matched donor, HLA-matched haploidentical stem-cell transplantation was performed on November 5, 2020, after signing a fully informed consent form. The following conditioning regimens were administered: fludarabine 30 mg/m^2^ daily from day -10 to day -6; total antithymocyte globulin (ATG) 10 mg/kg, separately given from day -7 to day -4; cyclophosphamide total 120 mg/kg, separately given from day -5 to day -2; mycophenolate mofetil 250 mg/m^2^ Bid, cyclosporine A 2–5 mg/kg per day with continuous intravenous administration (optimal concentration 250–350 ng/mL), methotrexate 10 mg/m^2^ on day + 1, + 3, and + 6 were used to prevent GVHD. Trimethoprim and sulfamethoxazole (TMP/SMX), ganciclovir, and posaconazole were utilized to prevent *Pneumocystis carinii* pneumonia, and cytomegalovirus and fungal infections, respectively. Ursodeoxycholic acid and alprostadil were employed to prevent SOS, and allopurinol was used for hyperuricemia prevention.

**Fig. 1:**
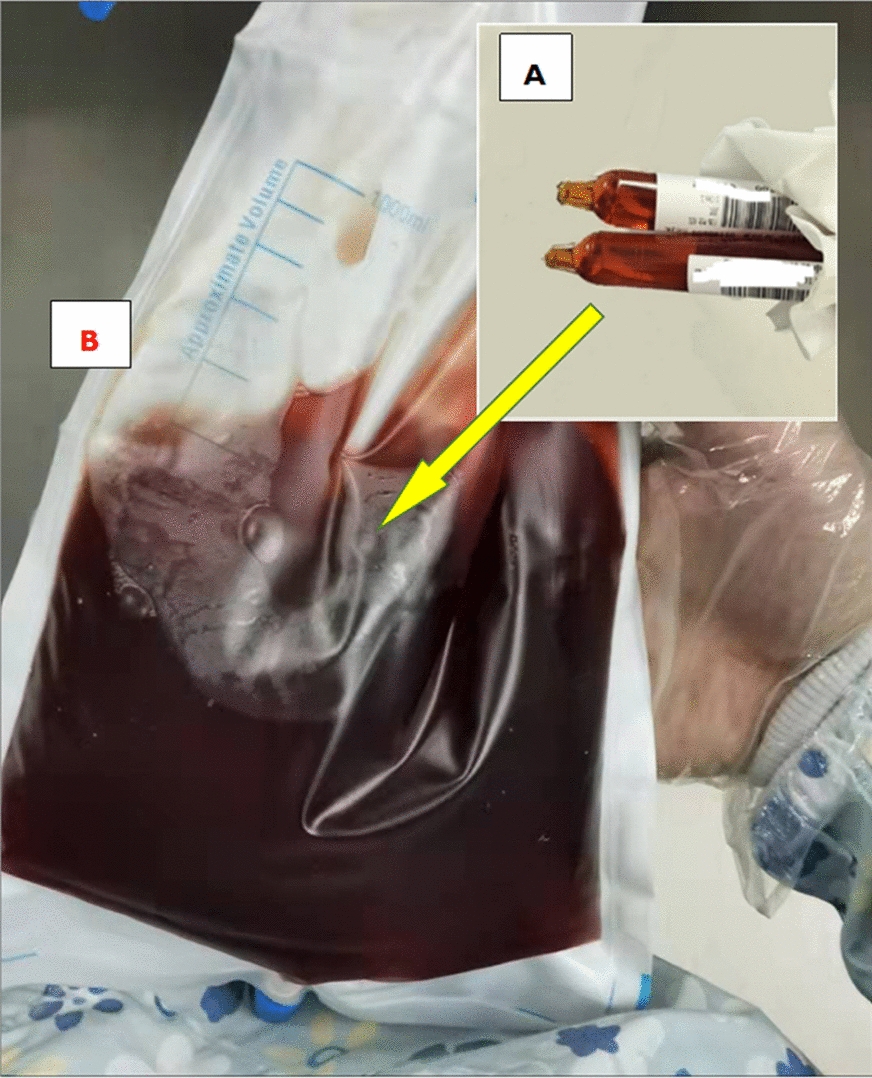
Changes in urine color. 1**A**: Shown in brown; 1**B**: shown in cola

**Fig. 2:**
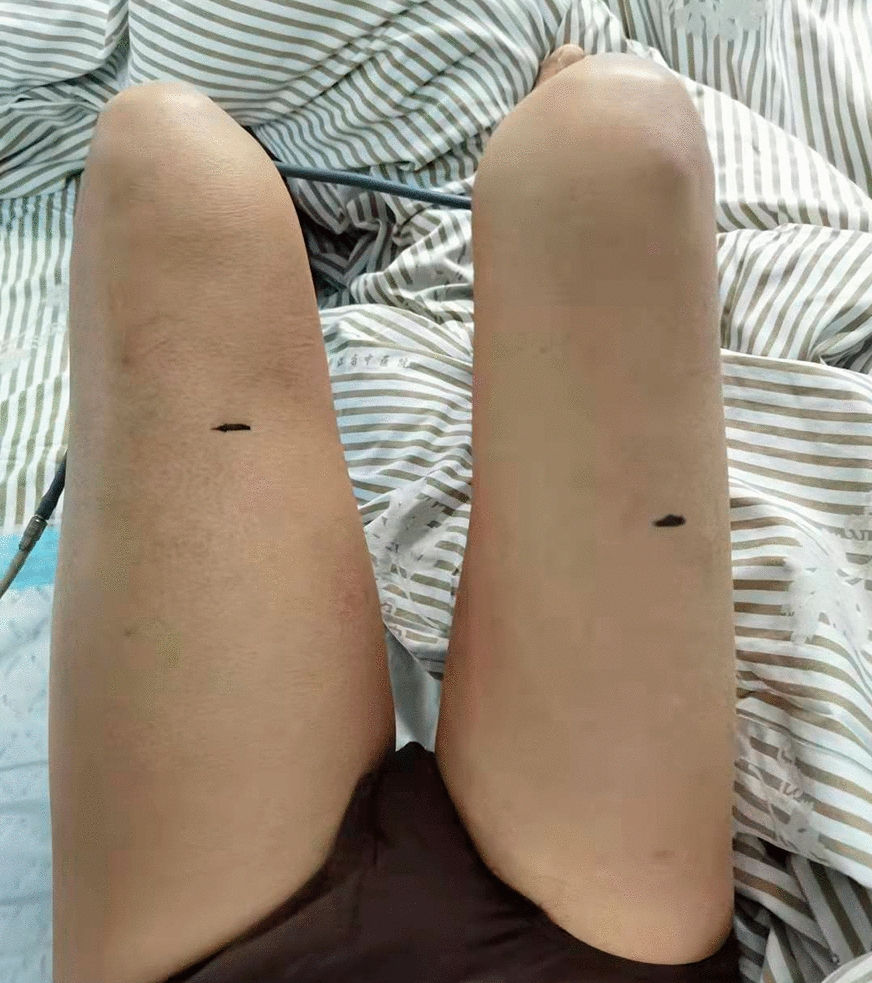
Swelling of the left thigh. The circumference 10 cm above patella was 41 cm on the left compared to that was 35.5 cm on the right; and the circumference 10 cm below patella was 32.5 cm on the left compared to that was 29.5 cm on the right

**Fig. 3:**
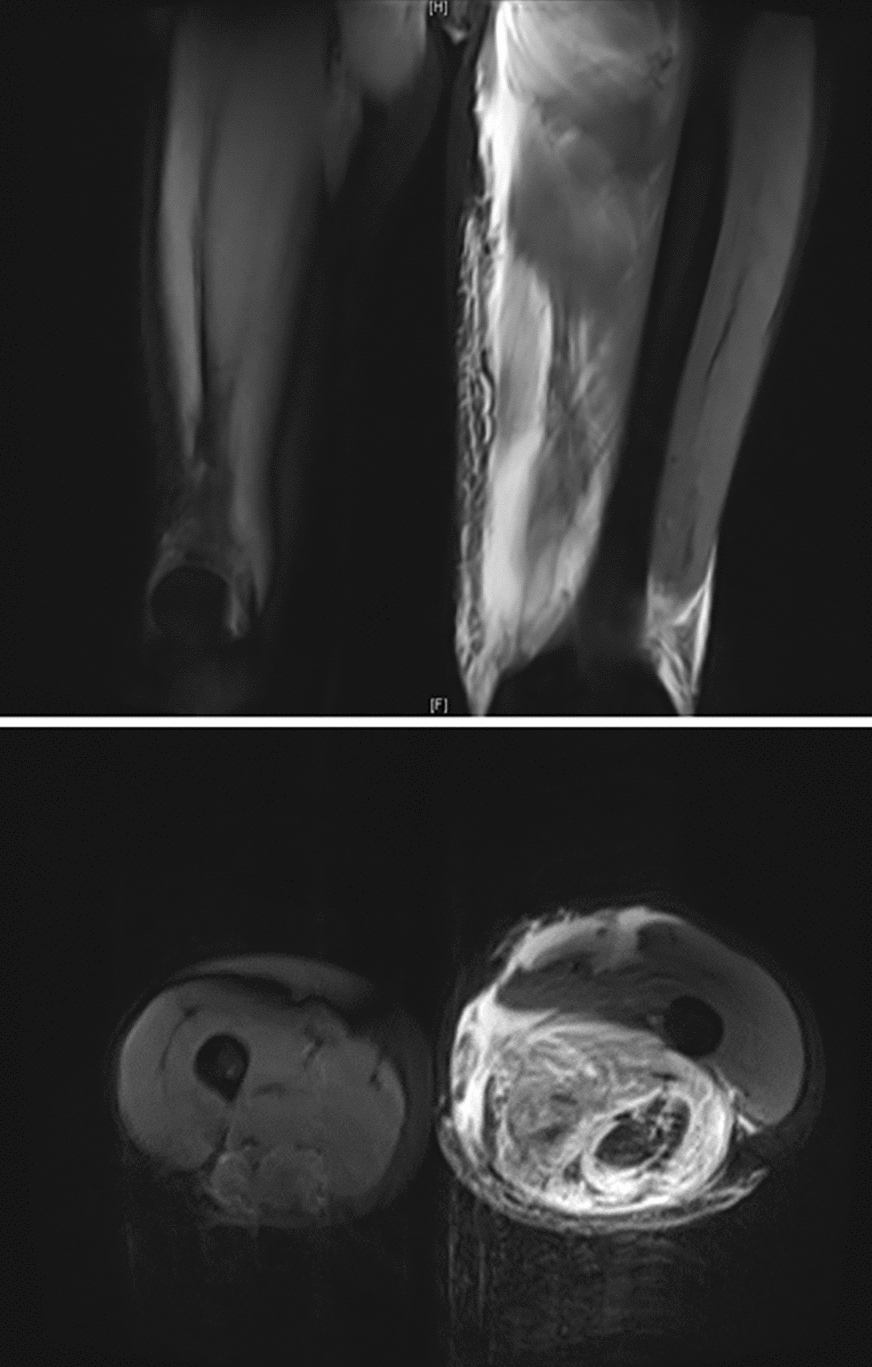
Magnetic resonance imaging (MRI) changes of the left thigh. The posterior and anteromedial muscles of the left thigh (gluteus maximus, rectus femoris, adductor longus, adductor brevis, and adductor magnus) and subcutaneous showed with fat exudative changes, and the interstitial space became blurred

Seven days after the donor stem-cell transfusion, at 1:40 AM, the patient complained of pain in the left inner thigh due to uneven force and muscle pulling when urinating. Various analgesic drugs were used with no beneficial effect. Two hours later, his body temperature rose to 37.6 °C. The blood test showed the following results: WBC 0.1 × 10^9^/L, ANC 0 × 10^9^/L, HB 62 g/L, PLT 28 × 10^9^/L, and C-reactive protein of 6.43 mg/L. Procalcitonin was 0.321 ng/mL. Serum inflammation cytokines: IL-6, 920.16 pg/mL and IL-10, 53.28 pg/mL; the others were within the normal range. Two hours later, his body temperature further increased to 38.6 °C. Considering the possibility of infection, meropenem was used. At 10:00 AM, the patient developed chest tightness, and laboratory tests showed that his creatine kinase (CK) was 500 U/L (38–174 U/L) and lactate dehydrogenase (LDH) was 273 U/L. He was given more hydration and alkalization for renal protection. Two hours later, the patient's symptoms did not significantly alleviate. The urine became darker (Fig. 1A). The muscle dissolution-related indicators were further checked, considering the diagnosis of rhabdomyolysis, but already 8 h had passed since his first complaint. The results showed that D-dimer was 1630 ng/mL (1–600 ng/mL) and myoglobin > 500 ng/mL. Urine routine parameters were: occult blood + , protein +  + , and red blood cells 16.5/μL. His symptoms worsened and the thigh became more swollen and painful (Fig. 2). Then, he underwent magnetic resonance imaging (MRI) at 3:30 PM, the result of which revealed exudation at the anterior and posterior muscle and subcutaneous fat of the left thigh (Fig. 3). AR was confirmed with atypical increased serum CK. At that time, the urine became darker (similar to soy sauce; Fig. 1B). Despite the aggressive supportive therapy, the patient’s condition continued to worsen within the next 2 h, with D-dimer of 2250 ng/mL, AST 196 U/L, CK 852 U/L, CK-MB 299.7 U/L, LDH 2393 U/L, creatinine (CREA) 188 μmol/L, and urea nitrogen 20.9 mmol/L. Five days after his death, the whole-exome sequencing results (peripheral blood and oral mucosal specimen) confirmed that the patient had had a germline missense mutation in SCN4A I 1545V and ACTN3 R577X.

## Discussion

Rhabdomyolysis is a disease caused by the rapid breakdown of skeletal muscle fibers and the release of intracellular components into the systemic circulation. The typical feature is the significantly increased serum CK activity (more than 5 times the normal level) [5]. Mild rhabdomyolysis manifests no muscular symptoms but only an increase in the serum CK. Some patients with severe rhabdomyolysis may have the classic triad of muscle pain, weakness, and dark urine. AR was commonly induced by trauma, exercise, muscle hypoxia, genetic defects, infection, body temperature changes, electrolyte disorders, drugs, etc. [6–8].

Approximately 10%-40% of the patients with rhabdomyolysis develop acute kidney injury, which may dramatically increase the mortality rate to nearly 80% [9, 10]. Our careful review of the total number of 11 cases (including ours) of rhabdomyolysis discovered during HSCT so far and showed that 7/11 patients had acute kidney injury, of which 4 died, 3 improved after hydration and alkalization [11–20]. Early fluid resuscitation for rhabdomyolysis treatment has been reported to restore the renal perfusion and is considered the main preventive treatment for acute kidney injury (AKI). If AKI developed, it is more recommended to start CRRT with a continuous high flux filter [21].

We analyzed 10 previously reported cases of rhabdomyolysis which occurred during HSCT, 6 of which were drug-induced, mostly by statins [17, 18], Cyclosporine A (CsA) [11, 17, 18], and high-dose chemotherapy [13, 16, 20]. Rhabdomyolysis caused by statins is usually concentration-dependent. Drugs that inhibit the metabolism of statins substantially increase the risk of statin-induced rhabdomyolysis [22]. CsA competitively inhibits atorvastatin metabolism through CYP3A4 [23, 24]. Posaconazole is also an inhibitor of CYP3A4, which increase the risk of rhabdomyolysis if used with statins together [25, 26]. Atorvastatin, a new type of molecular modulator, which can target a variety of immune cells, is thought to reduce the mortality of acute GVHD and the incidence of chronic GVHD [27, 28]. Volin, et al. [11] reported that when fluconazole is used in combination with CsA, the concentration of CsA increase to more than 1500 ng/mL, which induces rhabdomyolysis. Excessive CsA alone was reported to induce rhabdomyolysis[19]. Our patient used atorvastatin for a short period of time (from day -10 to day -1) during HSCT as GHVD prophylaxis. However, rhabdomyolysis occurred 8 days after the atorvastatin withdrawal. During this period, his CsA concentration was tested every 3 days and was within the range 154.8–219.9 ng/mL. The role of atorvastatin and CsA in the development of rhabdomyolysis is still unclear. However, we should cautiously concern the necessity and safety of the application of atorvastatin for GVHD prophylaxis in HSCT. In addition, high-dose chemotherapy was also found to induce rhabdomyolysis. Shima, et al. [16] reported that their patient, who received cyclophosphamide chemotherapy at a triple dose, developed rhabdomyolysis after 19 h; CK increased to more than 100 times over the normal value within 2 days. Hoshi, et al. [13] also reported that large doses of chemotherapeutic drugs damaged the kidney during pretreatment, leading to rhabdomyolysis. Our patient used cyclophosphamide during pretreatment, which might have also been implicated in the development of the disease. The role of steroids in rhabdomyolysis development is unclear, but some investigations revealed that myopathy caused by steroids tends to be more severe and with a worse prognosis [19, 29], whereas others supported the notion that methylprednisolone could be a salvage regimen for rhabdomyolysis [30, 31].

In addition to drugs, infection is another common cause of rhabdomyolysis after transplantation [8, 12, 14, 15]. Our patient was in the stage of agranulocytosis, immunosuppressed and thus susceptible to infection. His body temperature was within the normal range before rhabdomyolysis. On the day of the rhabdomyolysis onset, the patient had fever with increased IL-6, IL-10, and PCT. Although the blood bacterial culture, EB virus, cytomegalovirus, and mycotoxin test results were negative, we could not completely rule out the possibility of infection.

It is worth noting that in this case, the patient had a trauma as a clear cause for the condition. He complained of muscle pull and pain before the disease onset, followed by swelling of the thighs. Seemingly, the trauma was not serious and would hardly cause rhabdomyolysis. However, the positive result obtained of his whole-exome sequencing revealed that he had a germline SCN4A (exon24: c. A4633G: p.I1545V) and ACTN3 (exon15:c.C1729T: p.R577X) heterozygous mutations. In the gnomAD database, the frequency of the SCN4A I1545V missense point mutation and ACTN3 heterozygous mutations is 8.008/10^5^ and 0.539, respectively. Asaf, et al. [6] found that over 40% of the patients with rhabdomyolysis had myolysis-related gene mutations. Previous reports suggest that mutations in ACADVL, ANO5, CPT2, DMD, DYSF, FKRP, HADHA, PGM1, LPIN1, PYGM, and RYR1 genes may cause rhabdomyolysis. Moreover, AGL, CAPN3, CNBP, DMPK, MAGT1, ACADM, SCN4A, SGCA, SGCG, SMPD1, and TANGO2 were found to increase the susceptibility to rhabdomyolysis [32]. The SCN4A gene is expressed in skeletal muscle and encodes a member of the sodium channel alpha subunit gene family. It has been reported that SCN4A mutations are expressed in approximately 86% of muscle channelopathies [33]. Its mutations can cause skeletal muscle channel diseases, including paramyotonia congenita, hypokalemic periodic paralysis, hyperkalemic periodic paralysis, congenital myasthenic syndrome, and rarely rhabdomyolysis [6, 7, 34]. The ACTN3 gene encodes a member of the α-actin-binding protein gene family, which is expressed mainly in the skeletal muscles and serves as a structural component of the Z-line of the skeletal muscles. Studies have shown that although the ACTN3 R577X mutation does not cause a disease, it is closely related to exercise-induced rhabdomyolysis, increasing the possibility of exertional rhabdomyolysis [33, 35]. In addition, another randomized controlled trial showed that the RX genotype was more susceptible to muscle injuries in sports than the RR genotype [36]. In our case, since the admission day, the patient was protected by a laminar flow bed and was directly moved into the transplantation chamber. The patient spent all 2 months in bed, without properly exercising. Although the patient in this case did not over-exercise, the long-term lack of exercise muscles is stretched instantly, which might have also led to rapid decomposition of the skeletal muscle fibers.

The major issue in our case was that we had not instantly diagnosed rhabdomyolysis and initiated CRRT as our first consideration, even at a normal CREA level. The main reason for this misleading initial diagnosis was that the CK level (500 U/L) at the early stage in our case was not as extremely high as earlier reported, even at the end of the patient's life (Tables 1 and 2).

**Table 1:** Changes of rhabdomyolysis and biochemistry tests throughout the progress

Time	CK (U/L, 38–174)	CK-MB (U/L, 0–25)	LDH (U/L, 109–245)	Mb (ng/mL, 0–107)	AST (U/L, 0–40)	CREA (μmol/L, 59–104)	D-dimer (ng/mL, 1–600)
The day before	**/**	**/**	**/**	**/**	10	83	**/**
05:00 AM	123	17.7	183	/	10	90	/
10:00 AM	500	28.3	273	/	21	/	/
13:00 PM	/	/	/	> 500	/		1630
18:00 PM	852	299.7	2393	> 500	/196	188	2250

*CK* creatine kinase, *Mb* myoglobin, *AST* aspartate transaminase, *CREA* creatinine

**Table 2:** Case review on rhabdomyolysis during hematopoietic stem cell transplantation

Case	Study	Years/gender	Primary disease	Conditioning regimen	Transplant	Time	Suspected cause	Symptom	Creatine kinase	AKI	Therapy	Fate
1	Volin, et al., 1990^11^	27/M	CML	Cyclophosphamide, TBI	MSDT	+ 3 month	CsA, corticosteroids, fluconazole	Grand mal seizure, bilateral lower extremity weakness, pain, dark red urine	81,000 U/L	Yes	Peritoneal dialysis	Died
2	Maruyama, et al.,1994^12^	17/F	Ki-1 lymphoma	Melphalan, etoposide and TBI	PBSCT	+ 23 day	CMV infection	Severe muscle weakness, muscle pain	110 mU/mL (< 25 mU/mL)	Yes (Cr = 4.2 mg/dl)	Hydration, alkalization	Survive
3	Hoshi, et al.,1999^13^	38/M	CC	Ifosfamide, carboplatin, etoposide	Auto-HSCT	0 day	HDC, pretreatment renal dysfunction, ifosfamide, sedatives	Dyspnea, hemoptysis, dark red urine	6150 IU/L	Yes	Hemodialysis	Died (respiratory failure)
4	Pugliese, et al.,2000^14^	Unknown	Breast cancer	Cyclophosphamide	Auto-HSCT	+ 7 day	Vancomycin	Severe muscle weakness	1756 U/L	No	Hydration, alkalization	Survive
5	Rossi, et al.,2000^15^	16/M	ATL	TBI, etoposide, cyclophosphamide	MSDT	+ 11 day	ABCD	Muscular hypertonus, trismus, severe muscular pain	21,730 U/L	No	Intensive care unit	Survive
6	Shima, et al.,2002^16^	47/F	ATLL	TBI, cyclophosphamide	NO	Pre-transplant period	High-dose cyclophosphamide	Generalized convulsions, muscle fatigue, severe acidosis	34,863 IU/L	No	Hydration, alkalization	Survive
7	Tong, et al.,2005^17^	66/M	MM	TBI, cyclophosphamide	Auto-HSCT, MSDT	+ 22 day	CsA, simvastatin	Bilateral lower extremity weakness, pain	29,253 U/L	Yes (Cr = 2 mg/dl)	Hydration, alkalization	Survive
8	Vives, et al.,2008^18^	54/M	AML	Fludarabine, busulfan	MSDT	+ 1 month	Simvastatin, CsA, risperidone	Pelvic muscle weakness, severe muscular pain	88 370 U/L	Yes (Cr = 4.81 mg/dl)	Hydration, alkalization	Survive
9	Jiang, et al.,2016^19^	41/F	CML	Busulfan, cyclophosphamide	MSDT	+ 55 day	Infection, GVHD, metabolic disorders, CsA, methylprednisolone	Anasarca and muscle tenderness	1614 μg/L(25–200 μg/L)	Yes	Unknown	Died
10	Sokolova, et al.,2017^20^	21/M	GCT	Unknown	Auto-HSCT	+ 12 day	Paclitaxel, ifosfamide, carboplatin, etoposide	Bilateral leg pain	30,841 IU/L	No	Hydration, alkalization	Survive
11	Our case	55/M	HAAA	Fludarabine, ATG, cyclophosphamide	Haplo-HSCT	+ 7 day	Gene mutation, muscle strain, Infection, TMP/SMX, atorvastatin	Muscle pain, dark urine, swelling of the left thigh	852 U/L	Yes	Hydration, alkalization	Died

*CML* chronic myeloid leukemia, *CC* choriocarcinoma, *ATL* acute T-cell lymphoblastic leukemia, *ATLL* adult T-cell leukemia/lymphoma, *MM* multiple myeloma, *AML* acute myelogenous leukemia, *GCT* germ cell tumor, *HAAA* hepatitis-associated aplastic anemia, *MSDT* matched sibling donor transplantation, *PBSCT* peripheral blood stem cell transplantation, *Auto-HSCT* autologous hematopoietic stem cell transplantation, *Haplo-HSCT* haploidentical hematopoietic stem cell transplantation, *TBI* total body irradiation, *HDC* high-dose chemotherapy, *CsA* cyclosporine A, *CMV* cytomegalovirus, *ABCD* amphotericin B colloidal dispersion, *AKI* acute kidney injury

In conclusion, AR is a rare but threatening complication during HSCT, especially in cases with kidney dysfunction. The CK level may not truly and completely reflect the severity and prognosis for cases with localized lesion, such as ours, with lysis limited to the affected thigh. We suggest that genetic analysis should be performed for better understanding the pathological changes of AR during HSCT, especially for patient with bone marrow failure disease.

## Data Availability

The data used and/or analyzed during the current study are available from the corresponding author upon a reasonable request.
